# Visualization of unstained DNA nanostructures with advanced in-focus phase contrast TEM techniques

**DOI:** 10.1038/s41598-019-43687-5

**Published:** 2019-05-10

**Authors:** Yoones Kabiri, Raimond B. G. Ravelli, Tibor Lehnert, Haoyuan Qi, Allard J. Katan, Natascha Roest, Ute Kaiser, Cees Dekker, Peter J. Peters, Henny Zandbergen

**Affiliations:** 1 Kavli institute of nanoscience Delft, Delft, 2629HZ The Netherlands; 2 The Maastricht Multimodal Molecular Imaging Institute, Universiteitssingel 50, Maastricht, 6229 ER The Netherlands; 3 Materialwissenschaftliche Elektronenmikroskopie, Universität Ulm, Albert-Einstein-Allee 11, 89081 Ulm, Germany

**Keywords:** Single-molecule biophysics, Cryoelectron microscopy, DNA nanostructures, DNA

## Abstract

Over the last few years, tremendous progress has been made in visualizing biologically important macromolecules using transmission electron microscopy (TEM) and understanding their structure-function relation. Yet, despite the importance of DNA in all forms of life, TEM visualization of individual DNA molecules in its native unlabeled form has remained extremely challenging. Here, we present high-contrast images of unstained single-layer DNA nanostructures that were obtained using advanced in-focus phase contrast TEM techniques. These include sub-Ångstrom low voltage electron microscopy (SALVE), the use of a volta-potential phase plate (VPP), and dark-field (DF) microscopy. We discuss the advantages and drawbacks of these techniques for broad applications in structural biology and materials science.

## Introduction

Although TEM imaging of DNA, in its native unstained form, is crucial for various applications across life sciences, it has remained extremely difficult to obtain such images. The challenges of DNA imaging with TEM are indeed manifold. Unstained DNA has only been made visible if freely suspended, i.e., without carbon support, and success in these experiments was limited to specific DNA structures such as DNA bundles or fibers^1–6^. When DNA is deposited onto commercial carbon membranes in dry condition, no good contrast can be achieved. Unfortunately, reducing the carbon thickness to increase the DNA contrast is not trivial due to difficulties in manufacturing and handling of delicate carbon membranes, as well as due to non-conductive properties of amorphous carbon below 4 nm thickness^7^, which strongly deteriorates the TEM imaging. Although the superior mechanical and electrical properties of graphene was conceived to provide a viable solution, the hydrophobic interaction between DNA and graphene has proven to be a major obstacle for imaging^8^.

**Figure 1:**
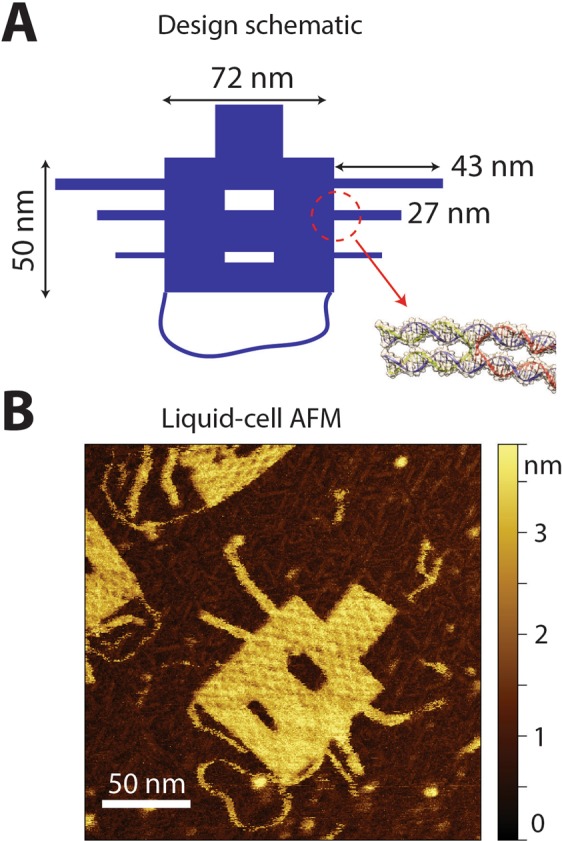
Single-layer DNA origami nanoplates as an innovative microscopy sample for single particle analysis. (**A**) Schematic of the 2D DNA origami design. The origami nanostructure contains various DNA features with different lengths and widths including symmetric side arms, cavities inside the main rectangle, and a floppy dsDNA loop at the bottom. The bottom-right inset illustrates the DNA helices equivalent. See Methods for the full details of the origami design. (**B**) Liquid-cell atomic force microscopy (AFM) image of an origami nanoplate on mica. Note that the bottom dsDNA loop as well as the single dsDNA arm (third from the top) depict a large flexibility. The high-resolution liquid-cell AFM image resolves Holliday junctions that are clearly distinguishable within the origami plate.

In this work, we aim to image unstained DNA nanostructures via modifications in the electron optics instead of addressing the sample preparation technicalities. We therefore choose the easiest route of sample preparation, which is support on commercially-available carbon membranes (i.e., not on delicate thin carbon supports) under dry conditions (i.e., not cryo-frozen^9^). This sample preparation route is thus very reproducible as well as accessible for every TEM lab. For convenient and reliable evaluation of double stranded (ds) DNA contrast, we utilize a single-layer DNA origami nanostructure (Fig. 1). The use of a two-dimensional DNA origami is an innovative and very useful approach because the specific shape of the origami structure allows reliable and convenient evaluation of dsDNA contrast. In addition, it enables the application of single particle analysis (SPA) to a purely nucleic acid structure, instead of, for example, using DNA-bound protein structures. Imaging multi-layered three-dimensional DNA origami structures is not pursued in this report since we aim for visualization of DNA at the single-helix thickness.

Electron optics in TEM have seen multiple improvements in recent years. While the conventional TEM (CTEM) is an ideal tool for imaging materials science samples with atomic resolution, it performs poorly for visualization of radiation-sensitive biological molecules such as DNA. Therefore, in order to increase the contrast, electron microscopists have tried to develop phase contrast techniques under low-dose conditions with the help of phase plates or low-voltage TEM. However, various technological issues have impeded this goal. For example, a commercial phase plate has only been available since 2015, namely the VPP^10^. Previous generations of phase plates including the well-known Zernike-type were difficult to fabricate, align, and integrate into a SPA workflow. In the case of low voltage microscopy, the significant deterioration of resolution caused by the chromatic aberration (C_c_) was the major limiting factor.

**Figure 2:**
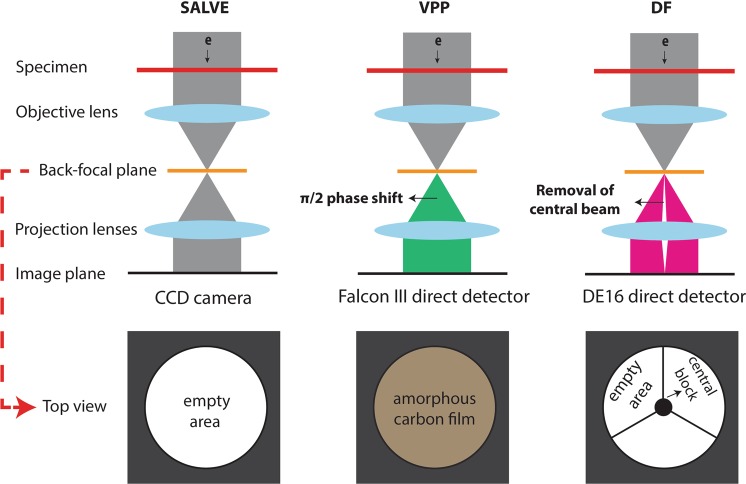
Schematics of in-focus phase contrast TEM techniques. The objective aperture design in the back-focal plane of the microscope for each technique is shown enlarged at the bottom row. SALVE technique does not require any special objective aperture design at the back-focal plane, whereas for contrast enhancement of DNA at high kV, π/2 phase shift or removal of the un-scattered zero beam is needed for VPP and DF, respectively. See the literature for the contrast enhancement mechanisms^10,13,17^.

Here, we present high contrast TEM images of unstained DNA nanostructures onto commercial carbon membranes using advanced “in-focus” TEM techniques through manipulation of electron optics shown in Fig. 2. Whereas CTEM renders poor contrast, we find that a chromatic and spherical aberration corrected (Cc + Cs) SALVE (sub-Ångstrom low voltage electron) microscopy, or the cosine-type phase shift induced by the VPP technology, resulted in an overall visibility of the DNA nanostructures, without the need of labelling. This enabled the particle picking and class averaging algorithms in the SPA workflow. The SALVE images of the DNA origami extend its application to life science specimens, which makes the low-kV route an attractive approach for imaging of both materials science and biological specimens. Next to the small or flexible protein structures under cryo condition^11^, we have here demonstrated VPP application for imaging the non-water embedded unstained nucleic acids. Furthermore, we show the visibility of DNA origami using the non-linear phase contrast DF technique. Finally, we discuss the prospect of SALVE and VPP techniques in terms of in-focus SPA workflow.

## Results

### Visualization of unstained DNA origami is not well possible with defocused CTEM

We were unable to detect sufficient contrast of DNA origami plates supported on commercial carbon membranes using the normal CTEM at 200–300 kV acceleration voltages, even with large defocus values (up to 10 µm) for the objective lens and even when the micrographs were acquired by a direct detector camera. The poor visibility of unstained 2D DNA origami on the commercial carbon membranes can be attributed to, on one hand, the low scattering of single DNA helix compared to relatively thick carbon support, and on the other hand, a large suppression of low-spatial frequencies in CTEM (Supporting Information (SI), Fig. S1), that are essential for particle edge detection and the overall visibility of the weak phase objects. Electron optical imperfections such as spherical aberration together with objective lens defocus were historically used as rather constructive ways to convert the low-frequency phase components of the exit wave into intensity modulations, though associated with severe damping of the higher resolution fringes (SI, Fig. S1). Nevertheless, this defocused CTEM approach could not render sufficient contrast to the origami plates supported on thick carbon membranes. Furthermore, acquisition of a good dataset in cryo-EM was not possible (exchanging the amorphous carbon substrate with amorphous ice). This could be attributed to the intrinsic floppy nature of the 2D DNA origami designs or the interfacial effects (air/water interface) during plunge-freezing step of cryo-EM sample preparation.

Taken all together, we thus note that the *de facto* defocused phase contrast method that is widely employed in the cryo-EM structure determination of proteins remains extremely challenging for contrast enhancement in our case study for non-water embedded DNA, which indeed makes the unstained single-layer DNA nanostructures one the most difficult samples to probe.

### Low-kV (80 kV) CTEM facilitates the DNA detection but results in blurry images

**Figure 3:**
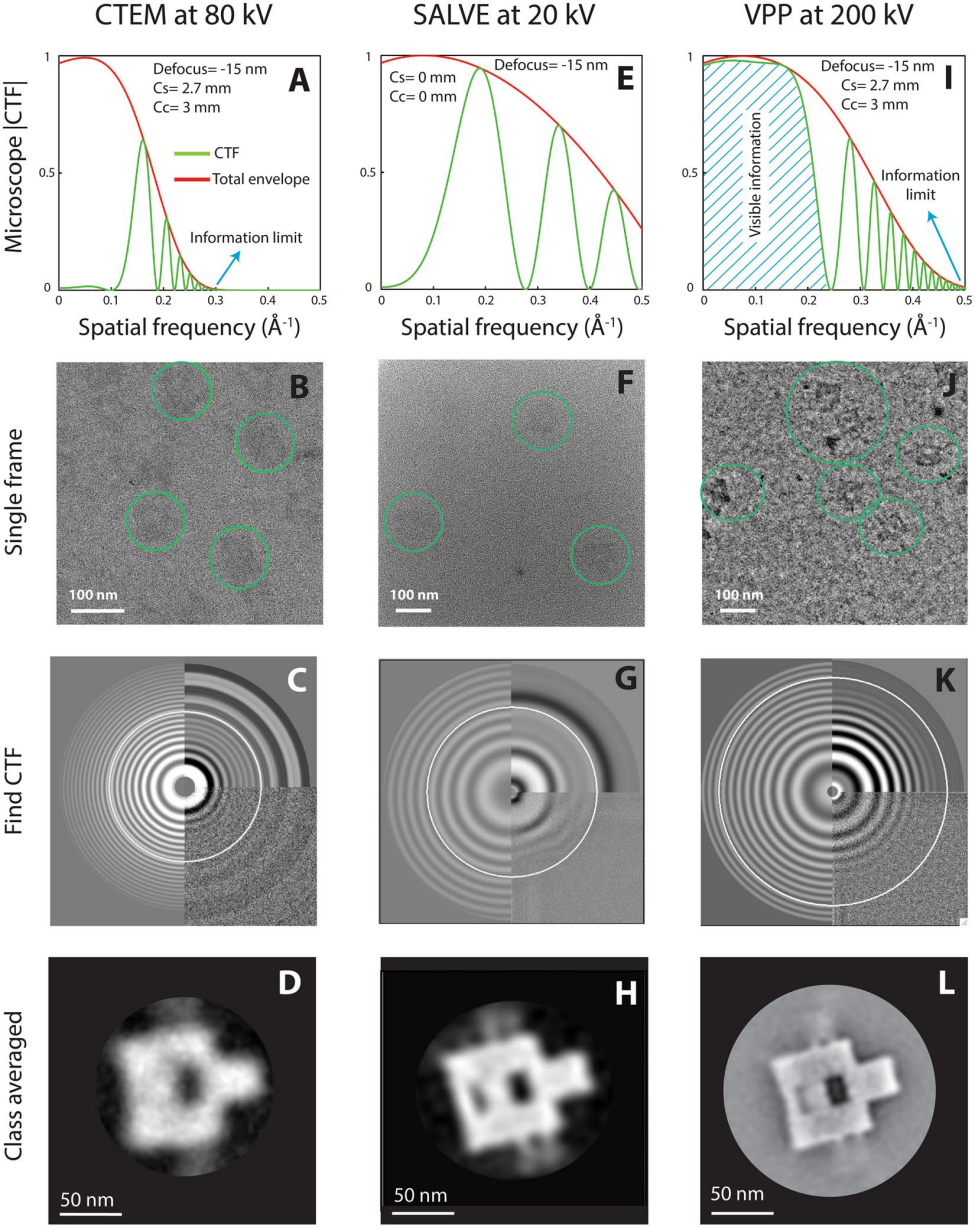
SPA results for imaging unstained DNA origami nanoplates supported on commercial carbon films. The figure compares three techniques: Column (**A**–**D**) shows results for 80 kV CTEM; column (**E**–**H**) for 20 kV SALVE microscope with Cs + Cc correction; and column (**I**–**L**) for VPP at 200 kV without Cs and Cc correction. (**A**,**E**,**I**) Theoretical CTF plots at near focus (green curves). The red curves indicate the total envelopes (spatial and temporal). We observe a significant resolution drop at 80 kV for CTEM due to pronounced Cs aberration, but do not observe this in the SALVE microscope. After implementing a VPP in a CTEM microscope, it is evident that the low spatial frequencies are hugely enhanced (the dashed area in panel I). We consider 0.1 amplitude contrast in all the CTF plots. (**B**,**F**,**J**) Single frame acquisitions. Sufficient contrast is achieved in each technique for enabling the particle picking and consequently SPA. Noticeably, the VPP depicts remarkable contrast in single frames. (**C**,**G**,**K**) Processed CTFs of micrographs. (**D**,**H**,**L**) SPA reconstructions. The CTEM reconstruction results in a blurry image due to presence of Cs aberration. Excitingly, the two novel approaches, SALVE and VPP, can alternatively be used to render sufficient contrast as well as good resolution for DNA imaging.

In order to increase the DNA contrast in CTEM, one remedy is to decrease the acceleration voltage, ideally down to 80 kV, because considerable increase in scattering cross section (both elastic and inelastic) is expected, which consequently facilitates phase contrast imaging. Reducing the high voltage from 300 kV in our Cs-corrected Titan to 80 kV within the non-Cs-corrected Arctica microscope, shifts the contrast transfer function (CTF) peaks towards the low-frequency components in the frequency spectrum (Fig. 3A). This is also indeed accompanied by a different performance of the detector as well as yielding a different amplitude contrast. Using this approach, we were able to detect a sufficient amount of contrast in single-frame acquisitions (Fig. 3B) at 80 kV acceleration voltage, which enabled the particle picking and consequently SPA. Figure 3D shows the 2D class-averaged image after CTF correction. We observe that the side arms of the origami, each having a 4 nm width, as well as the small cavity inside the rectangle (4 nm × 19.2 nm) are either severely blurry or hidden. This low-resolution reconstruction indicates the importance of Cs-correction in order to obtain high-resolution images. Prior to reconstruction, low-resolution outcome could also be predicted based on the CTF plot in Fig. 3A, where a considerable decrease in information limit is seen, down to 3.3 Å compared to 2 Å at 200 kV operation voltage of the Arctica microscope (SI, Fig. S1). In the next section, we proceed to correct for the aberrations.

### Cc + Cs correction at low-kV (20 kV, SALVE) strongly improves the contrast and resolution

Low-kV (i.e. 80 kV down to 20 kV) phase contrast electron microscopy was historically abandoned since the chromatic aberration of the objective lens severely deteriorated the resolution^12^. With the elimination of chromatic aberration, the resolution substantially improves at 20 kV (Fig. 3E, where we see pronounced low-frequency transfer as well as an improved information limit beyond 2 Angstrom). Recently, atomic resolution at 20 kV was successfully realized within the framework of the SALVE project^13^. This is a remarkable step towards studying sensitive samples below 80 kV prone to knock-on damage like graphene^13^. However, hydrogen-containing specimens are still challenging to image^14^. To probe what is possible, we thus imaged the DNA origami plates with the Cc + Cs corrected SALVE microscope to remove the severe delocalization effects at low-kV and at the same time obtain enough contrast for visualization of unstained DNA.

We find that the DNA origami reconstruction using the low-kV dataset provides excellent contrast (see Fig. 3H). Strikingly, we observe that the detailed DNA structural features such as the smaller cavity and the single DNA helices become visible using class averaging of a relatively small number of manually acquired frames (~100 micrographs). Increased total scattering cross section at 20 kV, together with enhanced low-frequency information transfer, boosts the phase contrast of the DNA. Specifically, Cc correction has an important role which enables focusing both the elastic and inelastic electrons into the same imaging plane and hence strengthening the contrast. Here, we presented first images of single-helix-thick unstained DNA nanostructures supported on commercial carbon supports at 20 kV obtained with the new Cs + Cs corrected technology. Our results extend SALVE microscope application for biomolecular imaging.

### Volta-potential phase plate substantially boosts the DNA contrast at high voltages

Although the SALVE microscope did provide pivotal results, the instrument availability is still very limited. Furthermore, various practical challenges should be overcome in terms of automation in data acquisition, cryo compatibility, and etc. If working at standard high kVs (100–300 kV) is desired (which is the norm in most TEM labs), one should tackle the historical CTF obstacle. One possible route to enhance the contrast of weak phase objects at high kV is to improve the low frequency transfer of the CTF, e.g., by changing the conventional sine-type (zero phase shift) into a cosine-type (i.e., π/2 phase shift). Then, a pronounced phase contrast at near-focus can become possible. Note, for example, the significant revival of the low-frequency components in the dashed area of Fig. 3I after inducing a 0.5 π phase shift. To realize this type of CTF performance, we employed a recent VPP technology in CTEM to probe the DNA contrast at 200 kV.

Importantly, we find that VPP micrographs display exceptionally good contrast for unstained DNA (Fig. 3J). We observe even the side arms of the DNA origami plates without class-averaging. Such VPP images imply that the low-spatial frequencies play crucial role in the overall visibility of the DNA nanostructures. Owing to the striking DNA contrast in single acquisitions, the class averaging of the origami plates could be achieved with only a handful (~2000) of particles. The reconstructed image (Fig. 3L) provides a detailed view of all the DNA spatial features that were incorporated in the origami design, e.g., the side arms (2 and 4 nm wide), and the cavities inside the rectangle (4 and 8 nm wide). We thus demonstrated the applicability of VPP at high kV, for imaging unstained single-layer flat nucleic acids on commercial carbon supports, resolving features down to the level of single dsDNA molecules.

Conjugating a VPP with CTEM without Cs correction at 200 kV was sufficient to obtain a good reconstruction of the origami plates. For our case study of nucleic acids, revival of low-frequency components in the frequency spectrum has proven to play a more significant role than the expensive aberrations correction. The versatility brought by VPP to be used in conjugation with a non-Cs-corrected microscope at high kV is indeed a tremendous advantage compared to other phase contrast techniques.

### Dark-field microscopy provides necessary contrast for DNA visualization at high voltages but is not suitable for SPA

**Figure 4:**
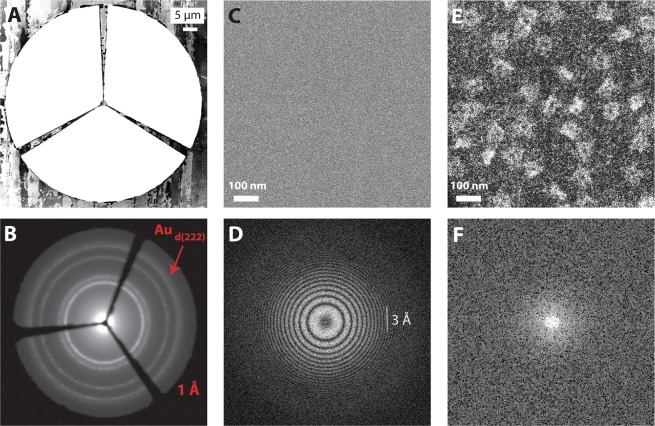
Dark-field visualization of unstained DNA nanostructures. (**A**) Fabrication of the delicate DF aperture by FIB milling on a 5-µm thick platinum foil. (**B**) Image of the aperture located in the diffraction plane. We see blocking of the zero beam, and a cutoff frequency of about 1 Angstrom (calibrated by a polycrystalline Au sample). (**C**,**E**) Bright- and dark-field images of the DNA nanostructures, respectively. We observe that the DNA structures are invisible in bright-field, but they do appear visibly in the dark-field image after insertion of the DF aperture. (**D**,**F**) Fourier transform of high-resolution images in panels C and E, respectively. The absence of carbon Thon rings in panel (F) is attributed to removal of central beam (linear interaction is absent). Thon rings in panel D are seen to extend beyond 3 Angstrom, which indicates a good performance of the DE-16 direct detector camera.

Alternatively, dark-field microscopy could be another approach for contrast enhancement at high kVs. The conventional DF and scanning TEM (STEM) techniques are the prevailing methods in materials science for studying inorganic samples. Since the diffracted beams in such inorganic samples are stronger (compared to biomolecules), the collection of only a fraction of diffracted beams suffices to form a DF image. However, in the case of single-DNA origami structures, a DF image can only be formed with high contrast after all scattered electrons are gathered in the wide-field TEM mode. For this reason, we fabricated a “Mercedes star” aperture (Fig. 4A) to remove the unscattered central beam while letting virtually all the scattered beams pass through. The aperture consists of a very delicate ion-milled central disc of ~1 µm in diameter, and a cut-off frequency of 1 Angstrom (Fig. 4B), see the Methods section of the manuscript for details on the aperture fabrication and imaging.

Interestingly, we find that utilization of a DF aperture in conjugation with a direct electron camera leads to DNA visualization at 300 kV (Fig. 4E). Whereas these DF acquisitions exhibit high contrast, the counterpart images of DNA nanoplates in bright-field TEM mode remain invisible (Fig. 4C). Note that acquisition and illumination parameters are the same in Fig. 4C,E, except that the DF aperture is inserted and aligned in the back-focal plane in Fig. 4E. We previously imaged the positively stained origami structures with DF using a conventional CCD camera^8^. But, the unstained images (current work) were not optimal until we replaced the CCD with a direct electron device (DE-16 Direct Electron, California). The is related to the lower noise and higher detective quantum efficiency (DQE) of direct electron cameras, which facilitates the contrast in DF.

With the DF technique, we intended to probe for an easier replacement for SALVE and VPP for facile visualization of weak-phase objects at high kVs. The DF technique is indeed easy and helpful for sample screening, especially because the DF aperture can be easily integrated into the objective aperture holder of any TEM machine without high costs. One should note that the size of DF aperture would differ substantially at low kVs due to expansion of diffraction space. Moreover, the image contrast would be affected by the residual Cs at the low kVs^8^. Therefore, DF imaging below 300 kV was not desired or conducted. Since alignment of the DF aperture should be carried out before every DF acquisition, and due to absence of Thon rings (Fig. 4F) that are critical for defocus measurement and data acquisition, it is unlikely that DF would become a routine in the SPA workflow^15^, until further software and automation obstacles are overcome.

## Discussion

We presented high contrast images of unstained single-layer DNA nanostructures on commercial carbon membranes using advanced in-focus phase contrast TEM techniques. We notice significant contrast enhancement at low acceleration voltages such as 20 kV, which was possible via Cs + Cc aberration correction (in the SALVE approach), or at high acceleration voltages such as 300 kV after inducing π/2 phase shift (in the VPP approach).

To understand and compare the suitability of SALVE and VPP for SPA in structural biology, one should consider the effect of defocus. Indeed, phase contrast methods that deliver both contrast and resolution at in-focus conditions (<±100 nm) are highly desired since they provide computational advantages over the prevailing defocused-based CTEM approaches in terms of data processing^16^. SALVE and VPP techniques differ from CTEM since they can be fully exploited at the in-focus conditions. Therefore, we discuss the effect of defocus on the expected resolution for these two techniques. Note that our definition of expected resolution for the in-focus condition differs substantially from the one mostly used in materials science, i.e., that is the information limit (as indicated in Fig. 3A). We define the resolution based on the 0.5 amplitude threshold in the CTF plot without any zero-crossing at the low-frequency domain of the CTF^16^.

**Figure 5:**
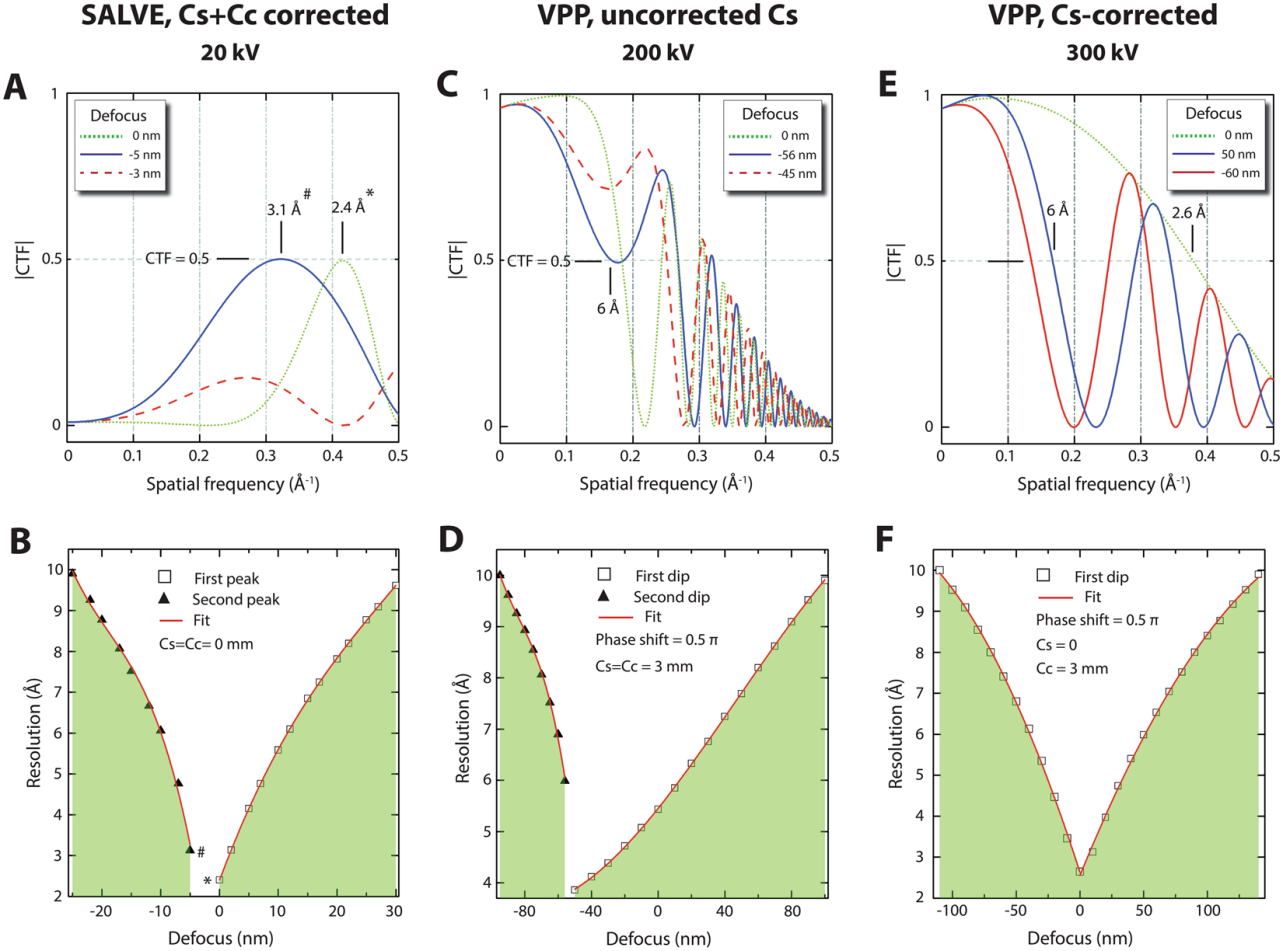
Effect of focus on the expected resolution for VPP and SALVE techniques. (**A**,**C**,**E**) Representative CTF plots at different focus values for SALVE, non-corrected VPP and Cs-corrected VPP, respectively. The resolution criterion is 0.5 amplitude threshold in each CTF plot. (**B**,**D**,**F**) Expected resolution vs defocus for each technique mentioned above. The data points are extracted according to CTF plots (see, for example, the * and # in panels A and B). The green highlighted areas are prohibited in terms of 0.5 CTF criterion. These analyses indicate that the defocus range for SALVE technique is very narrow, which makes the data acquisition very challenging. On the contrary, VPP depicts a greater defocus tolerance at comparable resolution, hence facilitating the SPA workflow. We also note that removing the Cs could further improve the VPP performance in terms of resolution and permitted defocus range.

Figure 5 depicts the effect of defocus on the expected resolution for SALVE and VPP. Let us start to investigate such an effect for the SALVE approach (Fig. 5A,B). Figure 5A shows SALVE CTF plots at three different defocus values. At zero focus (green dotted line), we expect a 2.4 Å resolution based on the |CTF = 0.5| criterion. This point is indicated as * in both panel A and B. Over-focusing (defocus >0) shifts this peak to the left-hand side of the frequency spectrum, where its amplitude reaches the |CTF = 0.5| threshold without any phase flipping at the low spatial frequencies, resulting in drop of resolution from 2.4 Å at zero focus to about 10 Å at +30 nm defocus. The right curve in Fig. 5B thus shows the resolution evolution for the defocus >0 illumination. On the contrary, for under-focus values (defocus <0), we observe that a second peak develops in the CTF plot (see the red dashed curve for −3 nm defocus). At −5 nm defocus, it fulfils the |CTF = 0.5| criterion at the resolution of 3.1 Å, indicated as # in both panel A and B. Greater under-focus values deteriorate the expected resolution from 3.1 Å at −5 nm defocus to 10 Å at −25 nm defocus (left curve in Fig. 5B depicting the resolution evolution for defocus <0 illumination). The green-highlighted area in Fig. 5B is restricted according to CTF performance, meaning that obtaining better resolution would not be possible. We note that the allowed defocus range at certain resolution is very narrow in the SALVE technique, which practically affects the SPA data collection.

For the VPP technique, similar analyses are provided in Fig. 5C-D. The major difference in VPP CTF is the cosine-type CTF. Note that the cut-on frequency is ignored in these plots and we consider complete 0.5 π phase shift performance for the volta-potential phase plate. We find that VPP allows for a greater acceptable defocus range, indicating that VPP is a superior technique than SALVE for SPA in terms of in-focus data collection. Figure 5E,F shows the effect of Cs correction on VPP. This CTF performance can nowadays be achieved with a commercial Titan Krios instrument equipped with VPP operating at 300 kV. We see that removing Cs in conjugation with a 0.5 π VPP phase plate increases the allowed defocus range at comparable resolution (compare with Fig. 5D). Moreover, the observed phase-flipping in the CTFs of SALVE and VPP following over- or under-focusing is absent in the Cs-corrected VPP, where we obtain a symmetric defocus-resolution plot in Fig. 5F.

TEM imaging of unstained nucleic acids opens up ample opportunities in life sciences. Studying label-free DNA-protein interactions, imaging the native chromatin structure, and imaging conjugated DNA nanostructures are just a few examples. More specifically, the 2D DNA origami, with its ample and diverse available sequences at its surface, provides a promising approach for biophysical assays such as probing sequence-specific protein interactions. It has been a long endeavor to develop TEM techniques for such applications. In the current work, we have shown that such images can be obtained using advanced in-focus phase contrast TEM techniques. Although our focus was on imaging nucleic acids, the insights provided by these techniques will be of further interest for broader applications in structural biology and materials science.

## Methods

### 2D DNA origami design

A 2D DNA origami was designed (Fig. 1A) in order to have the same scattering yield as in a single dsDNA molecule. The rectangular shape of the origami facilitates its detection on carbon membranes and enables particle picking and consequently SPA. The full details of the origami design and its characterization can be found elsewhere^8^. An additional side arm was incorporated in the design to have single DNA helix (~2 nm as in B-form DNA) extruding from the oligomer sequence within the main rectangle. The liquid-cell AFM data (Fig. 1B) showed that this arm was very flexible and therefore hard to resolve in the TEM class averaging.

### TEM sample preparation

The investigated samples were similar for all of the techniques discussed in this manuscript. They were unstained single-layer DNA origami (Fig. 1) deposited on commercially available TEM grids (Electron Microscopy Science, USA, 3–4 nm carbon supported on 5–6 nm formvar). 3 µL of purified origami sample with a concentration of 5 nM was drop casted on freshly glow-discharged TEM grids, and left to incubate for 2 minutes. Subsequently, the grids were thoroughly washed with Milli-Q water and gently blown dry with a nitrogen flow. Following this protocol, we achieve a high density and homogenous distribution of the DNA origami plates on TEM grids.

### SALVE data acquisition and processing

The Cc + Cs corrected SALVE microscope was operated at 20 kV at room temperature to image DNA origami. The aberration coefficients Cc and Cs were tuned to −10 µm and −20 µm, respectively. The microscope is equipped with a Ceta 16 M camera (FEI). The exposure time was set to 1 s and a dose rate of 61 e s^−1^ nm^−2^ was applied for the manually collected dataset. We used Lentzen conditions (Microsc. Microanal. 14, 16–26, 2008) for the defocus which is around 10 nm with fifth order spherical aberration of ~3 × 10^6^ nm in our case. After initial screening of the dataset, the low-kV collected dataset contained 108 images. The CTFs were fitted on the dose-weighted micrographs with Zhang’s Gctf (Kai Zhang, 2015), a GPU-accelerated program for real-time CTF determination and correction. The following parameters were used: spherical aberration −20 µm, voltage 20 kV, amplitude contrast 0.1, minimum resolution 40 Å, maximum resolution 4 Å, minimum defocus 1000 Å, maximum defocus 10000 Å (step size 500 Å). Additionally, the astigmatism was 500 Å, B-Factor of 300 Å and the additional validation option was used. After visual inspection, 92 CTF-corrected images were used for the selection of particles. Particle picking was carried out manually using the GPU-accelerated beta version of Relion 2.1 (Scheres, 2017), with a particle diameter of 1500 Å, pixel size 1.698 Å. Particles were extracted with a box size 1024, based on the particle diameter and pixel size. From the 92 images, 240 particles were extracted and were subjected to 2D-classification using the following parameters: 5 classes, with regularization parameter of 3, 25 iterations and a mask diameter of 1600 Å. After 2D classification, the particles were sorted and selected on a Zmas score of 0.8. These particles were again subjected to 2D classification (3 classes, regularization parameter 3, 25 iterations and a mask diameter of 1600 Å).

### VPP data acquisition and processing

The data was collected on an FEI Tecnai Arctica (FEI) cryogenic TEM, operated at 200 kV at liquid nitrogen temperature and equipped with a Falcon III detector (FEI). The data was acquired using the following parameters: magnification x53,000, 50 µm C2 aperture, spot size 5, 40.718% C2 lens, 35.129% diffraction lens, pixel size 1.97 Å, dose rate on the detector 47.7 electrons/pixel/s. The data was acquired by the automatically with EPU software (FEI). The phase plate was aligned to provide optimum phase shift performance. Exposure time 5 s, with 200 frames and 20 fractions per movie (10 frames/fraction). The periodicity of the phase plate was set to 50 exposures, with an activation time of 10 s. After initial screening of the dataset, the VPP collected dataset contained 1848 images. The movies were aligned using MotionCorr2. Parameters were 5 × 5 patches, dose-weighting 2 electrons/Å2, pixel size 1.97 Å and a b-factor of 100 was applied. The CTFs were fitted on the dose-weighted micrographs with Zhang’s Gctf (Kai Zhang, 2015). The following parameters were used: spherical aberration 2.7 mm, voltage 200 kV, amplitude contrast 0.1, minimum resolution 30 Å, maximum resolution 4 Å, minimum defocus 1000 Å, maximum defocus 10000 Å (step size 500 Å), minimum phase shift 20 degrees, maximum phase shift 160 degrees with a step size of 10 degrees, the astigmatism was 150 Å and the additional validation option was used. After visual inspection, 1720 CTF-corrected images were used for the selection of particles. Particle picking was carried out manually using the GPU-accelerated beta version of Relion 2.1 (Scheres, 2017), with a particle diameter of 1500 Å, pixel size 1.97 Å. Particles were extracted with a box size 1024, based on the particle diameter and pixel size. From the 1720 images, 2193 particles were extracted and were subjected to 2D-classification using the following parameters: 3 classes, with regularization parameter of 2, 25 iterations and a mask diameter of 1600 Å. After 2D classification, the particles were sorted and selected on a Zmas score of 0.8. These particles were again subjected to 2D classification (3 classes, regularization parameter 3, 25 iterations and a mask diameter of 1600 Å).

### Dark-field image acquisition

The DF images were acquired in a post-specimen Cs-corrected Titan microscope at room temperature. The DF aperture, which is positioned and aligned in the back-focal plane (Figs 2 and 4), was fabricated by a FEI Helios dual beam machine assisted by an automated CAD software for milling, see full details of our DF aperture fabrication and alignment procedures provided elsewhere^8^. Briefly, the microscope was initially aligned in bright-field mode to remove the Cs and axial aberrations such as astigmatism. The DF aperture was then inserted and aligned in the diffraction space. No change in astigmatism was observed after DF aperture insertion, tested via image sharpness of small (5 nm) Au nanoparticles (since the FFT of DF images lack Thon rings to correct for astigmatism). A direct electron camera (DE-16, Direct Electron, California) was employed to efficiently collect all the scattered electrons (Fig. S2).

### CTEM

The CTEM data were recorded with the same VPP microscope at liquid nitrogen temperature, though the phase plate capability was switched off and the operating voltage was tuned down to 80 kV. This microscope features uncorrected Cs and Cc values of 2.7 mm and 3 mm, respectively.

## Supplementary information

SI

## Data Availability

The raw data will be deposited in the Electron Microscopy Public Image Archive EMPIAR. Additional representative micrographs for each technique is shown in Fig. S3.
