# Supplementary material for: Visualization of unstained DNA nanostructures with advanced in-focus phase contrast TEM techniques
Source: Sci Rep. 2019 May 10;9:7218. doi: 10.1038/s41598-019-43687-5 (PMC6510773; doi:10.1038/s41598-019-43687-5)
Supplement: Supplementary file 1 — SI [file 41598_2019_43687_MOESM1_ESM.docx]

**Supporting information:**

Visualization of unstained DNA nanostructures with advanced in-focus phase contrast TEM techniques

*Yoones Kabiri ^1^, Raimond B. G. Ravelli ^2^, Tibor Lehnert ^3^, Haoyuan Qi ^3^, Allard J. Katan ^1^, Natascha Roest ^2^, Ute Kaiser ^3^, Cees Dekker ^1^, Peter J. Peters ^2*^, and Henny Zandbergen ^1*^*

1. *Kavli institute of nanoscience Delft, Delft 2629HZ, The Netherlands*
2. *The Maastricht Multimodal Molecular Imaging Institute, Universiteitssingel 50, Maastricht 6229 ER, The Netherlands*
3. *Materialwissenschaftliche Elektronenmikroskopie, Universität Ulm, Albert-Einstein-Allee 11, 89081 Ulm, Germany*

*^*^ Corresponding authors:* [*peter.peters@maastrichtuniversity.nl*](mailto:peter.peters@maastrichtuniversity.nl)*,* [*h.w.zandbergen@tudelft.nl*](mailto:h.w.zandbergen@tudelft.nl)


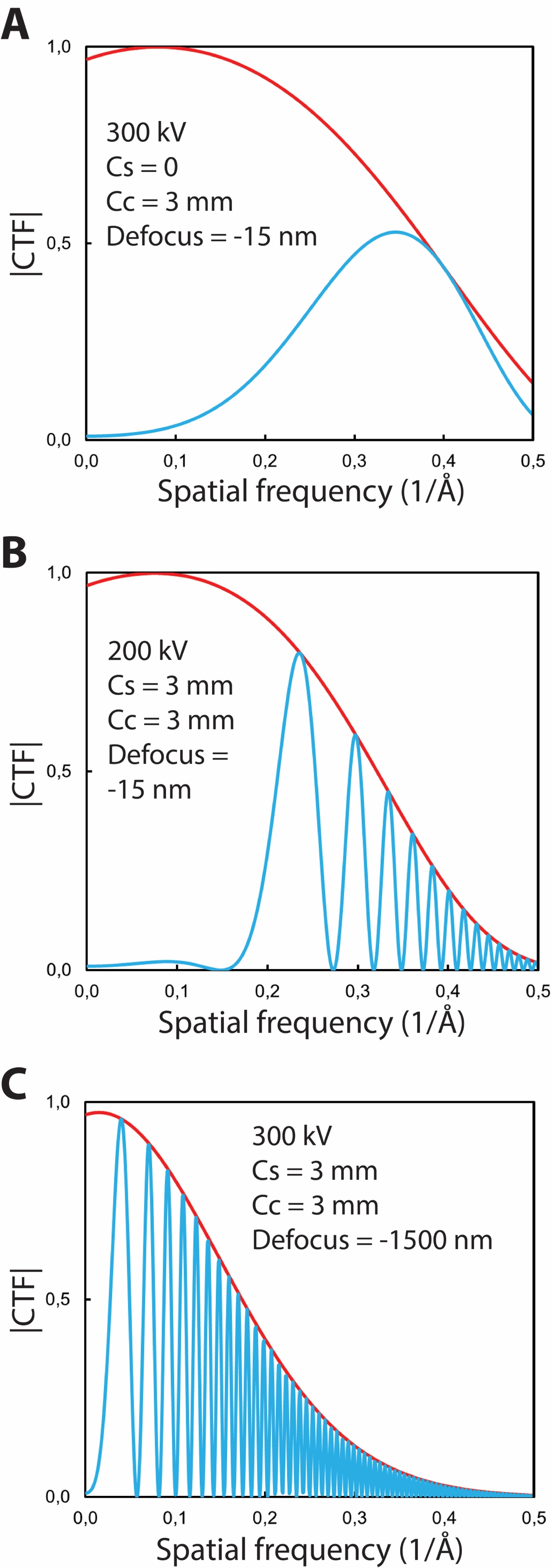


Figure S1. CTFs (blue curves) for (A) a Cs corrected Titan microscope operating at 300 kV (B) a non-corrected Arctica microscope operating at 200 kV (C) a non-corrected Titan microscope at 300 kV under strong defocus illumination. The red curves are the total envelope function (temporal and spatial).

**
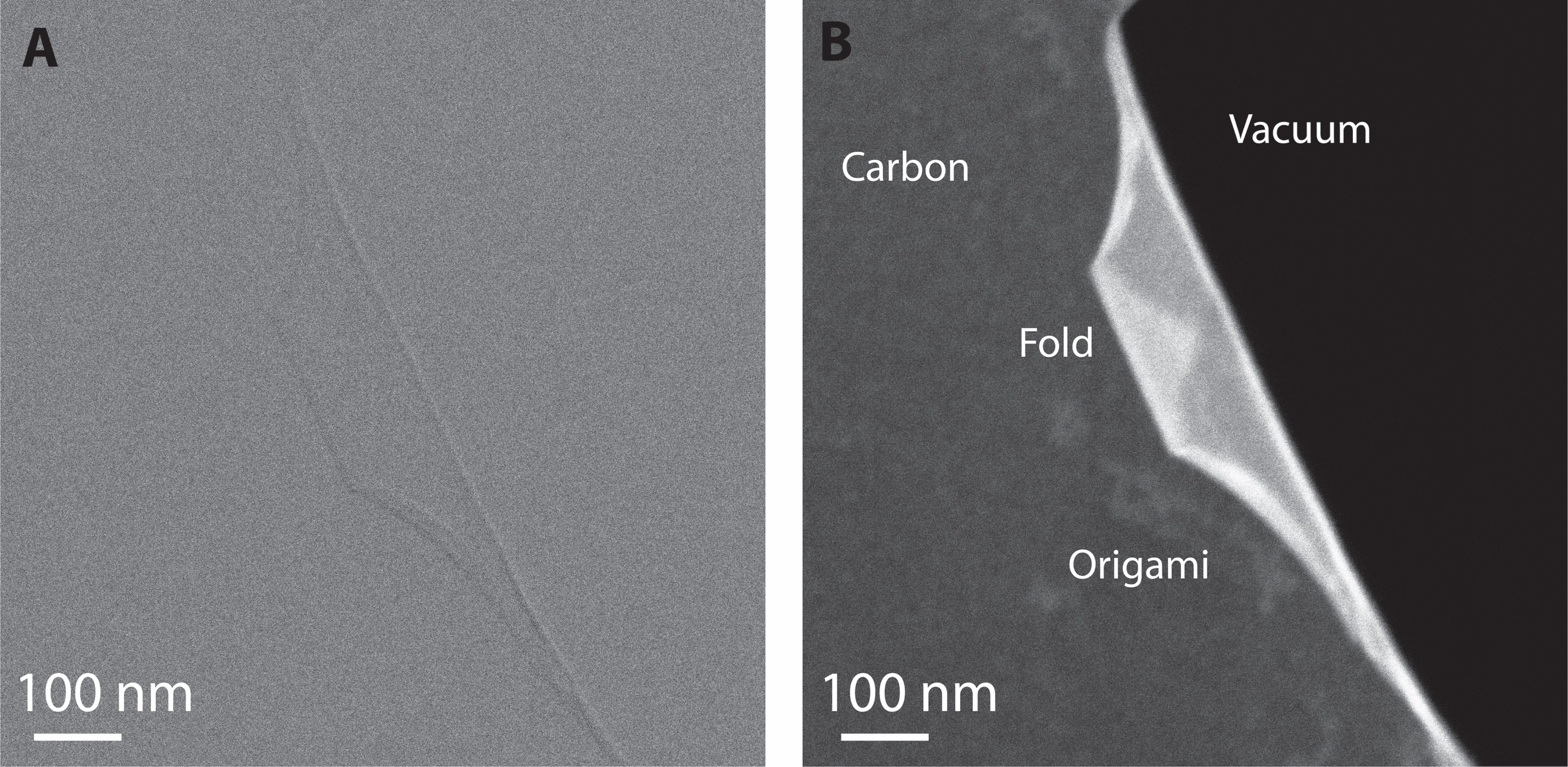
**

Figure S2. DF imaging performance in conjugation with DE-16 camera. (A) Bright-field and (B) DF image of a same area on the sample. Remarkable contrast enhancement is see after inserting DF aperture.

**
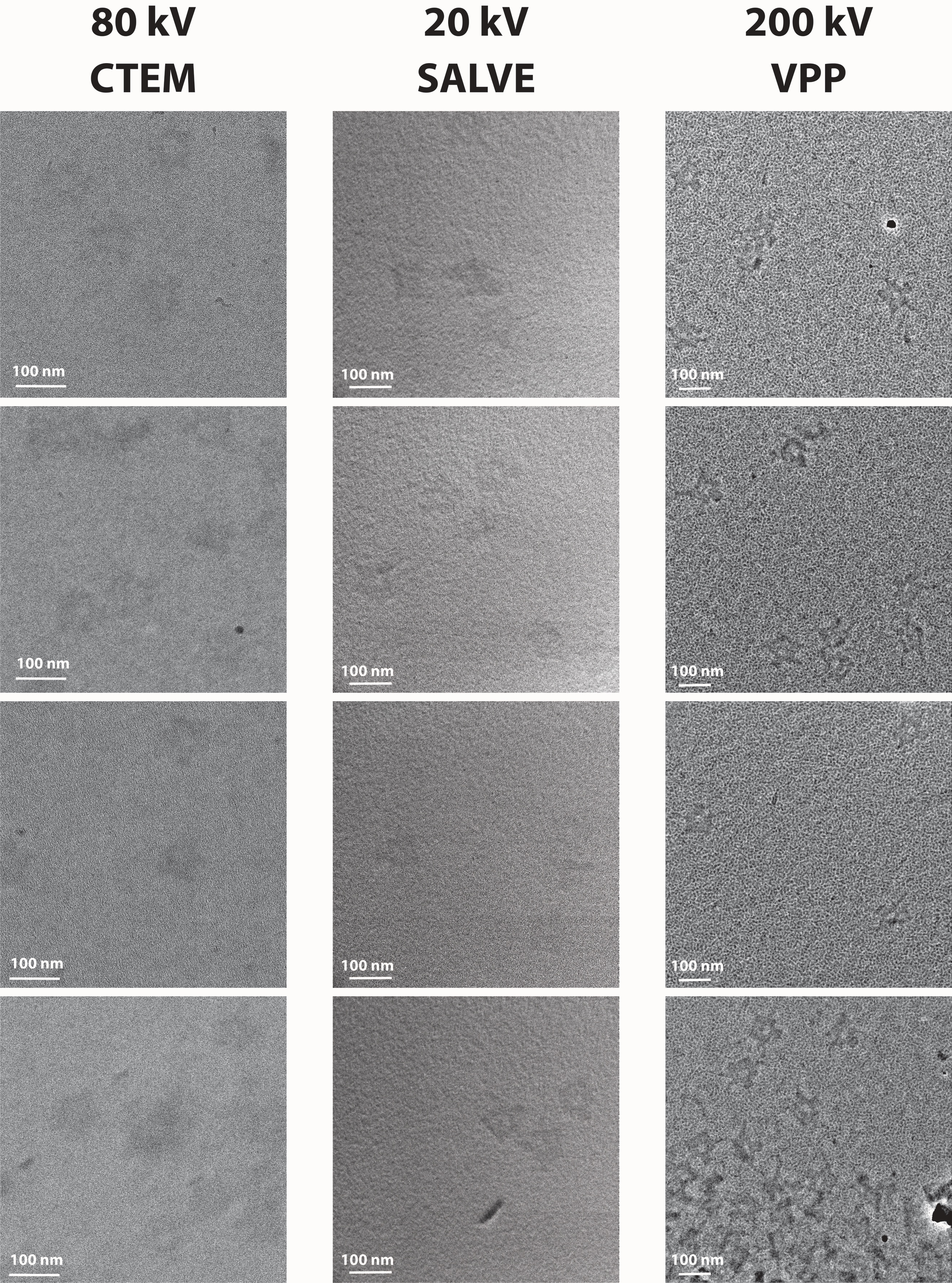
**

Figure S3. Additional exemplary micrographs. More data will be deposited in the Electron Microscopy Public Image Archive (EMPIAR).
